# *Acanthoatractis xinguensis* n. gen., n. sp. (Nematoda: Cosmocercoidea: Atractidae) parasite of yellow-spotted Amazon river turtle, *Podocnemis unifilis* Troschel (Testudines: Podocnemididae) in Brazilian Amazon

**DOI:** 10.1016/j.ijppaw.2024.100961

**Published:** 2024-07-02

**Authors:** Ronald Ferreira Jesus, Bianca Nandyara, Jeannie Nascimento dos Santos, Francisco Tiago de Vasconcelos Melo

**Affiliations:** Laboratory of Cellular Biology and Helminthology “Profa. Dra. Reinalda Marisa Lanfredi”, Institute of Biological Sciences, Federal University of Pará (UFPa), Av. Augusto Correa 01, Guamá, Belém, Pará, 66075-110, Brazil

**Keywords:** Brazilian Amazon, Atractidae, *Acanthoatractis xinguensis*, *Podocnemis unifilis*, Turtle nematode

## Abstract

Nematodes collected from the stomach of the yellow-spotted turtle *Podocnemis unifilis* Troschel, 1848 (Testudinidae) in the Brazilian state of Pará are assigned to a new genus, allocated to the family Atractidae (Cosmocerdoidea). *Acanthoatractis* n. gen. differs from all other genera of Atractidae based on the combination of the following morphological characters: cephalic extremity surrounded by eight bifurcated, wrench-shaped sclerotized structures arranged in a circle; oral opening encircled by two sclerotized pieces with pointed ends and a median portion with a pair of hooks; in males the larger (left) spicule is narrower in the middle third and the gubernaculum is absent. The type species, *Acanthoatractis xinguensis* n. gen., n. sp., has nine pairs of caudal papillae and a single papilla anterior to the cloacal lip. The new species is the seventh record of an atractid genus parasitizing *P. unifilis*.

## Highlights

• A new genus and species of atractids parasitizing freshwater turtles from the Amazon region.
• *Acanthoatractis* n. gen. differs from all other genera by the shape of sclerotized structures on the cephalic end.
• This is the 27th genus of the family Atractidae.
• This is the seventh record of an atractid genus parasitizing *Podocnemis unifilis*.

## Graphical abstract

**Figure:**
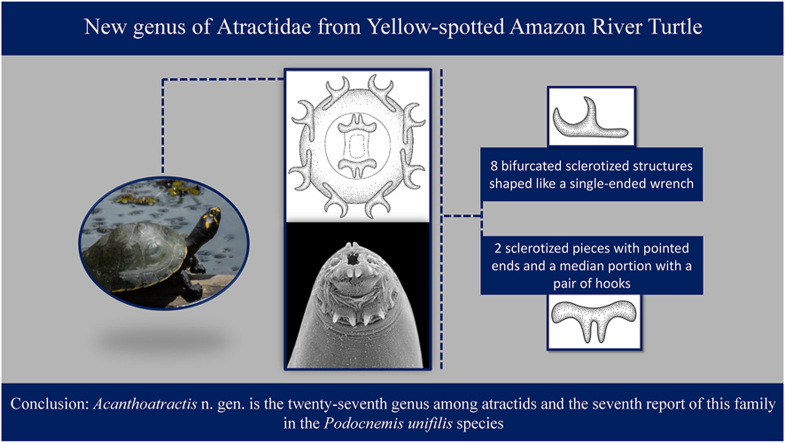

## 1 Introduction

Nematodes of Atractidae Railliet, 1917 (Travassos, 1919) are frequently found parasitizing fish, amphibians, reptiles, and mammals. Currently, this family accommodate 27 valid genera, namely: *Atractis* Dujardin, 1845, *Labiduris* Schneider, 1866, *Probstmayria* Ransom, 1907, *Crossocephalus* Railliet, 1909, *Cobboldina* Leiper, 1911, *Cyrtosomum* Gedoelst, 1919, *Rondonia* Travassos, 1920, *Leiperenia* Khalil, 1922, *Monhysterides* Baylis and Daubney, 1922, *Grassenema* Petter, 1959, *Paratractis* Sarmiento, 1959, *Nouvelnema* Petter, 1959, *Klossinemella* Costa, 1961, *Pseudatractis* Yamaguti, 1961, *Fitzsimmonsnema* Petter, 1966, *Orientatractis* Petter, 1966, *Pseudocyrtosomum* Gupta and Johri, 1987, *Buckleyatractis* Khalil and Gibbons, 1988, *Podocnematractis* Gibbons, Khalil and Marinkelle, 1995, *Diceronema* Gibbons, Knapp and Krecek, 1996, *Paraorientatractis* Gibbons, Khalil and Marinkelle, 1997, *Rhinoclemmysnema* Gibbons and Platt, 2006, *Pneumoatractis* Bursey, Reavill and Greiner, 2009, *Rhinoceronema* Mondal and Manna, 2013, *Hippopotamenema* Mondal and Manna, 2015 and *Vogtnema* Jesus et al., 2022.

*Podocnemis* Wagler, 1830 comprises freshwater turtles endemic to northern South America (Ferrara et al., 2017). To date, six atractid genera have been reported from *Podocnemis unifilis* Troschel, 1848, including *Buckleyatractis*, *Orientatractis*, *Paraorientatractis*, *Paratractis*, *Pneumoatractis*, and *Podocnematractis* (Khalil and Gibbons, 1988; Gibbons et al., 1995, 1997; Bursey et al., 2009; Jesus et al., 2020). However, we found some atractid nematodes that we could not allocate to any already known genera of the family. Thus, we propose and describe a new genus and species based on light and scanning electron microscopy.

## 2 Materials and methods

Three specimens of *Podocnemis unifilis* were collected from the Xingu River (“Volta Grande” region), municipality of Vitória do Xingu, Pará, Brazil, during a survey of helminths in freshwater turtles. Hosts were anesthetized by injection of 2% ketamine and posteriorly euthanized by ketamine overdosage. The organs of the gastrointestinal tract (stomach, small and large intestines) were carefully removed, isolated in Petri dishes, and examined under a LEICA EZ4 stereomicroscope (Leica Microsystems, Wetzlar, Germany). Nematodes were washed in saline solution (NaCl 0.9%) and heat-killed in 70% ethanol. For morphological and morphometric analysis, the nematodes were cleared in 20% Aman's lactophenol, following a protocol adapted from Amato et al. (1991), and examined using an Olympus BX41 microscope (Olympus, Japan) with a drawing tube. Measurements are in micrometers unless otherwise indicated and are presented as the range followed by the mean in parentheses.

We selected six specimens (three males and three females) for scanning electron microscopy (SEM) analyses. The nematodes were post-fixed in 1% OsO_4_, dehydrated in a graded ethanol series (30–100%), critical point dried with CO_2_, and placed on aluminum stubs using carbon tape and sputter coated with gold/palladium. We analyzed the specimens under a Vega3 microscope (TESCAN, Brno, Czech Republic) with an acceleration voltage of 10 kV in the Laboratory of Cellular Structural Biology (LBE) at the Federal University of Pará (UFPa).

The type-material was deposited in the Invertebrate Collection of the Helminthological Collection of Oswaldo Cruz Institute (CHIOC), Brazil.

## 3 Results

### 3.1 *Acanthoatractis* Jesus and Melo n. gen

*Diagnosis*: Cosmocercoidea. Atractidae. Body medium-sized, tapering anteriorly, tail short and pointed with cuticle finely longitudinally striated. Lateral alae absent. Cephalic end surrounded by eight bifurcated sclerotized structures (two subdorsal pairs, two dorsolateral pairs, two subventral pairs, and two ventrolateral pairs) shaped like single-ended wrench, arranged in circle. These sclerotized structures equal, positioned on opposite sides in both transverse and longitudinal planes. Oral aperture small, terminal, and rectangular, surrounded by two sclerotized pieces (one ventral and one dorsal); each piece with pointed ends and median portion with pair of hooks; two large lateral amphids present. Esophagus divided into two parts; anterior esophagus (corpus) with distal bulb muscular, posterior esophagus (isthmus), ending in non-valvulated bulb; nerve ring encircling isthmus; excretory pore anterior to esophageal bulb. Male with nine pairs of caudal papillae; spicules unequal, only larger (left) one narrower in middle portion, lanceolate; gubernaculum absent. Female monodelphic, ovoviviparous with vulva close to anus.

Etymology: The name of the genus comes from the greek “acanth/o,” meaning spine or thorn, referring to the presence of spines on the cephalic end of the nematode.

### 3.2 *Acanthoatractis xinguensis* Jesus and Melo n. gen., n. sp.

#### 3.2.1 General description

Medium-sized nematodes, body cylindrical, tapering to both extremities. Males and females similar in length without lateral alae. Esophagus divided into defined corpus, elongated isthmus, and a bulb. Nerve ring situated in middle of esophagus, at beginning of isthmus; deirids small, located just below nerve ring. Excretory pore anterior to esophageal bulb, opening surrounded by radial cuticle striations; pore opening into large chamber with transverse striations. Posterior end with short, pointed tail, ventrally curved in both sexes.

#### 3.2.2 Male (based on holotype; 13 paratypes all adult males)

**Fig. 1:**
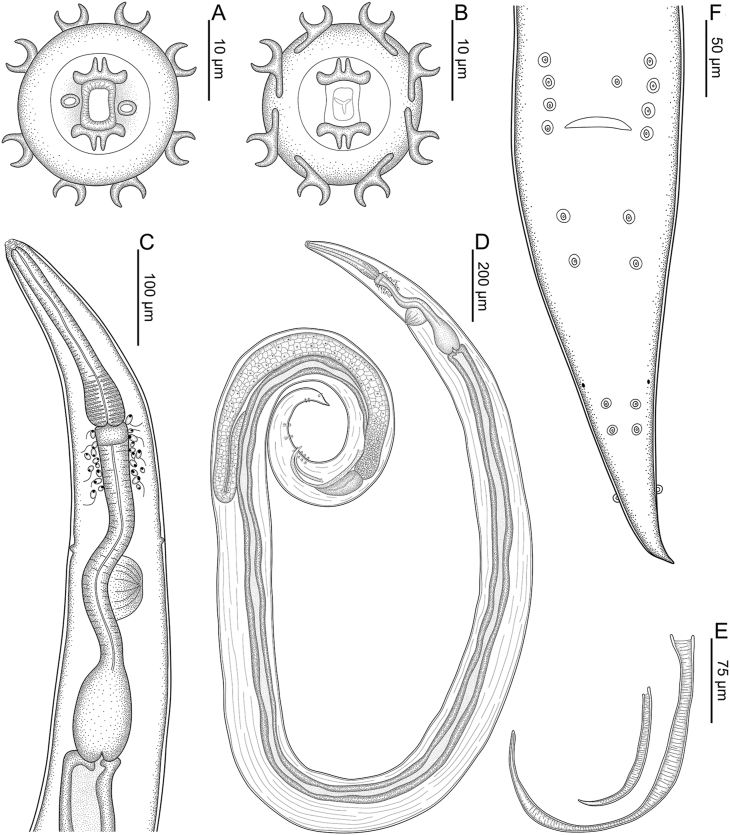
Line drawings of *Acanthoatractis xinguensis* n. gen., n. sp. (Male) **(A)** Cephalic extremity, apical view. **(B)** Cephalic extremity, apical view, highlighting the oral opening surrounded by sclerotized pieces and the distribution of open end wrench-shaped sclerotized structures. **(C)** Anterior extremity of body, ventral view. **(D)** Whole body, lateral view. **(E)** Details of spicules. **(F)** Posterior extremity of body, ventral view.

Total body length 4.21 (3.74–4.33) mm; width at esophago-intestinal junction 127 (97–127). Esophagus 548 (479–565) long with corpus, isthmus and bulb. Corpus 221 (198–221) long, distal bulb 58 (47–58) × 32 (29–36); isthmus including esophageal bulb 328 (266–357) long. Width of bulb 45 (38–52). Nerve ring, excretory pore and deirids at 240 (229–251), 411 (336–412) and 341 (326–367) respectively, from anterior end of body. Nine pairs of caudal papillae and one unpaired papillae anterior to cloacal lip: three subventral precloacal pairs, one subventral adcloacal pair, and five postcloacal pairs (first subventral, second subventral, third subventral, fourth subventral and fifth dorsolateral); phasmids ventrolateral, located between second and third pairs of postcloacal papillae 179 (177–209) (Fig. 1F; 3C). Spicules lanceolate, unequal, with transverse striations along its length, distal ends sharply pointed, larger (left) spicule narrower in middle third 343 (326–382) long, smaller (right) spicule 123 (110–149) long (Fig. 1E). Tail 317 (275–349) long (Fig. 1D–F; 3C).

#### 3.2.3 Female (based on allotype; 15 paratypes all adult females)

**Fig. 2:**
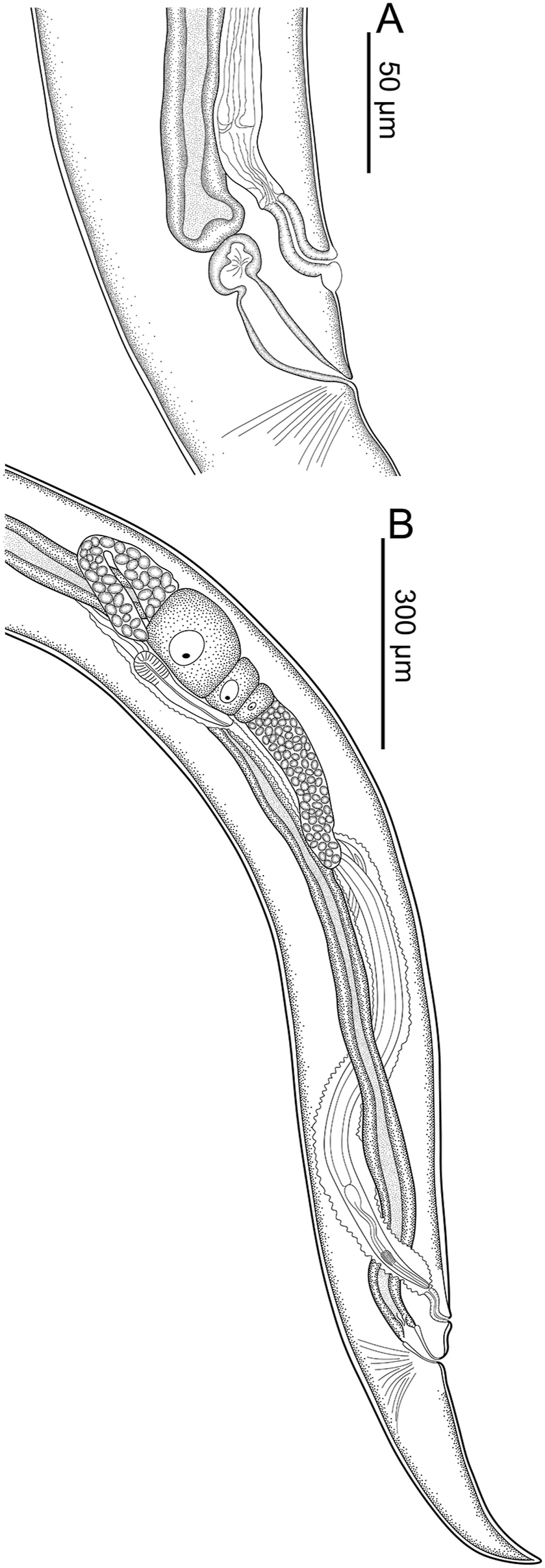
Line drawings of *Acanthoatractis xinguensis* n. gen., n. sp. (Female) **(A)** Posterior extremity of body, region of vulva and anus, lateral view. **(B)** Reproductive tract showing monodelphic uterus, lateral view.

**Fig. 3:**
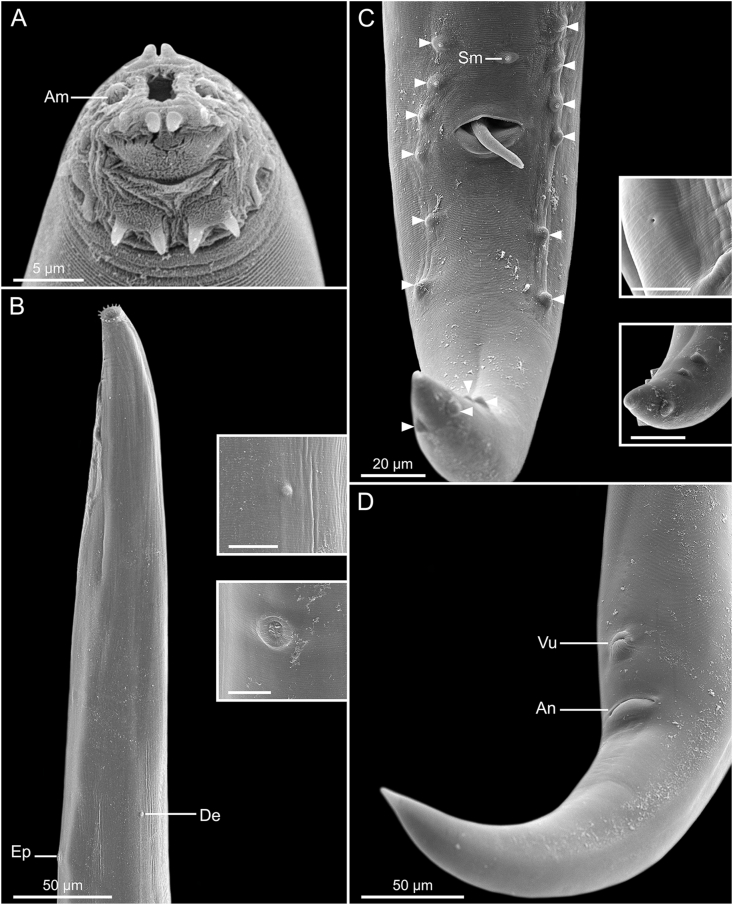
Scanning electron micrographs of *Acanthoatractis xinguensis* n. gen., n. sp. **(A)** Male, cephalic extremity, subapical view. **(B)** Anterior extremity of body, ventrolateral view. Inset: Detail of deirid, lateral view (Scale-bar: 10 μm); Detail of excretory pore, ventral view (Scale-bar: 10 μm). **(C)** Posterior extremity of male, ventrolateral, distribution of caudal papillae (arrowheads). Detail of phasmid, ventrolateral view (Scale-bar: 5 μm); Postcloacal papillae, ventrolateral view (Scale-bar: 10 μm). **(D)** Posterior extremity of female, ventrolateral view, lines indicate vulva and anus. Abbreviations: Amphid, Am; anus, An; deirid, De; excretory pore, Ep; single median papilla, Sm; vulva, Vu.

Total body length 4.01 (3.64–4.05) mm; body width at esophago-intestinal junction 126 (94–130). Esophagus 573 (485–573) long with corpus, isthmus and bulb. Corpus 217 (173–231) long, distal bulb 52 (44–56) × 30 (29–32); isthmus including esophageal bulb 356 (304–356) long. Width of bulb 54 (47–56). Nerve ring, excretory pore and deirids at 211 (211–259), 405 (344–405) and 371 (291–371) respectively, from anterior end of body. Phasmids located at 136 (132–140) from posterior end. Viviparous, vulva situated at 373 (301–373) from posterior end of body, from anus to vulva 47 (40–51). Muscular vagina anteriorly directed 41 (41–42). Monodelphic, prodelphic, uterus beginning in posterior flexion of vagina, directing anteriorly, containing one or two larvae (Fig. 2A and B; 3D). Tail 307 (256–332) long (Fig. 2B; 3D).

### 3.3 Taxonomic summary

**Superfamily Cosmocercoidea Railliet, 1916**.

**Family Atractidae Railliet, 1917 (Travassos, 1919**).

***Acanthoatractis xinguensis* Jesus and Melo n. gen., n. sp.** (Fig. 1, Fig. 2, Fig. 3)

*Type-host: Podocnemis unifilis* Troschel, 1848 (Chelonia: Testudinae).

*Type-locality:* Xingu River (“Volta Grande” region) (3°23′12.682″S 51°44′58.315″W), Municipality of Vitória do Xingu, Pará, Brazil.

*Type-specimens*: Holotype (CHIOC 39197a), allotype (CHIOC 39197b) and 28 paratypes (CHIOC 39197c-39197d) were deposited in the helminthological collection of Oswaldo Cruz Institute - Rio de Janeiro, Brazil.

*Site of infection:* Stomach.

*Prevalence*: 33% (1 infected/3 examined).

*ZooBank registration*: To comply with the regulations set out in article 8.5 of the amended 2012 version of the International Code of Zoological Nomenclature (ICZN, 2012), details of the new species have been submitted to ZooBank. The Life Science Identifier (LSID) for *Acanthoatractis xinguensis* n. gen., n. sp. Is urn: lsid: zoobank.org: act: ABE15A5D-A805-49BE-A544-DCBBDBA20EF6.

*Etymology:* The species was named after the Xingu River, the type locality.

## 4 Discussion

Jesus et al. (2022) proposed an identification key for Atractidae and listed 27 valid genera. Among these genera, *Labeonema* Puylaert, 1970 was included; however, molecular analyses indicated that the phylogenetic position of *Labeonema* is within Cosmocercidae Railliet, 1914 (see Saito et al., 2021). Therefore, in this study, we will consider 26 valid genera, listed in a table that includes type species, host, and biogeographic occurrence of Atractidae genera (see Table 1).

**Table 1:** Genus, type-species, host and Biogeographic occurrence of Atractidae (26 valid genera are included here and the new genus) around the world.

				
Genus	Type-species	Hosts	Biogeographic occurrence of species	Reference
				
***Acanthoatractis* n. gen**	***Acanthoatractis xinguensis* n. gen., n. sp.**	**Reptiles (Freshwater turtle)**	**Neotropical**	**This study**
*Atractis*	*Atractis dactyluris*	Fish and reptiles (Freshwater turtle, tortoise and Squamata)	Neotropical, Ethiopian, Oriental, Palaearctic	Dujardin (1845)
*Labiduris*	*Labiduris gulosa*	Reptiles (Tortoise)	Neotropical and Ethiopian	Schneider (1866)
*Probstmayria*	*Probstmayria vivipara*	Mammals	Neotropical	Ransom (1907)
*Crossocephalus*	*Crossocephalus viviparus*	Mammals	Oriental, Ethiopian	Railliet (1909)
*Cobboldina*	*Cobboldina vivipara*	Mammals	Oriental, Ethiopian	Leiper (1911)
*Cyrtosomum*	*Cyrtosomum scelopori*	Reptiles (Squamata)	Neotropical and Neartic	Gedoelst (1919)
*Rondonia*	*Rondonia rondoni*	Peixes and anfíbios	Neotropical and Australian	Travassos (1920)
*Leiperenia*	*Leiperenia leiperi*	Mammals	Ethiopian and oriental	Khalil (1922)
*Monhysterides*	*Monhysterides piscicola*	Fish and reptiles (Freshwater turtle)	Oriental and neotropical	Baylis and Daubney (1922)
*Grassenema*	*Grassenema procaviae*	Mammals	Ethiopian and oriental	Petter (1959)
*Paratractis*	*Paratractis hystrix*	Reptiles (Freshwater turtle)	Neotropical	Sarmiento (1959)
*Nouvelnema*	*Nouvelnema cyclophoron*	Mammals	Palaearctic	Petter (1959)
*Klossinemella*	*Klossinemella iheringi*	Fish and reptiles	Neotropical	Costa (1961)
*Pseudatractis*	*Pseudatractis testudinicola*	Reptiles (Freshwater turtle)	Oriental	Yamaguti (1961)
*Fitzsimmonsnema*	*Fitzsimmonsnema reptiliae*	Reptiles (Tortoise)	Ethiopian	Petter (1966)
*Orientatractis*	*Orientatractis levanhoai*	Fish, amphibians and reptiles (Freshwater turtle and tortoise)	Neotropical, Oriental, Ethiopian and Australian	Petter (1966)
*Pseudocyrtosomum*	*Pseudocyrtosomum lucknowensis*	Reptiles (Freshwater turtle)	Oriental	Gupta and Johri (1987)
*Buckleyatractis*	*Buckleyatractis marinkelli*	Reptiles (Freshwater turtle)	Neotropical	Khalil and Gibbons (1988)
*Podocnematractis*	*Podocnematractis ortleppi*	Reptiles (Freshwater turtle)	Neotropical	Gibbons et al. (1995)
*Diceronema*	*Diceronema versterae*	Mammals	Neotropical	Gibbons et al. (1996)
*Paraorientatractis*	*Paraorientatractis semiannulata*	Reptiles (Freshwater turtle)	Neotropical	Gibbons et al. (1997)
*Rhinoclemmysnema*	*Rhinoclemmysnema multilabiatum*	Reptiles (Freshwater turtle)	Neotropical	Gibbons and Platt (2006)
*Pneumoatractis*	*Pneumoatractis podocnemis*	Reptiles (Freshwater turtle)	Neotropical	Bursey et al. (2009)
*Rhinoceronema*	*Rhinoceronema unicornensis*	Mammals	Oriental	Mondal and Manna (2013)
*Hippopotamenema*	*Hippopotamenema aliporensis*	Mammals	Oriental	Mondal and Manna (2015)
*Vogtnema*	*Vogtnema asymmetrica*	Reptiles (Freshwater turtle)	Neotropical	Jesus et al. (2022)

We assign the specimens described here to Atractidae because the esophagus is divided into two distinct portions: an anterior, cylindrical portion (corpus) and a posterior, elongated portion (isthmus) ending in a bulb. Males lack a precloacal sucker in the caudal region and possess two unequal spicules. Females are monodelphic and ovoviviparous, with the vulva located in the posterior region of the body, near the anus (Anderson et al., 2009).

The number and morphology of lips and the presence/absence of sclerotized structures such as spines and hooks are the main characteristics that differentiate genera within the Atractidae (Jesus et al., 2022). Based on these characteristics, Atractidae can be divided into two groups of species; the first group comprises genera whose lips have associated sclerotized structures (hooks and/or spines), including *Klossinemella*, *Orientatractis, Crossocephalus*, *Paraorientatractis*, *Cobboldina*, and *Grassenema*. The second group includes genera without sclerotized structures at the cephalic end, namely *Nouvelnema*, with the oral opening surrounded by two lips, *Buckleyatractis*, *Labiduris*, *Paratractis*, *Pneumoatractis*, and *Pseudocyrtosomum* with three lips; *Cyrtosomum*, *Fitzsimmonsnema*, *Leiperenia*, *Monhysterides*, *Podocnematractis*, *Probstmayria*, *Pseudatractis*, *Rhinoceronema*, and *Rhinoclemmysnema*, which have six lips; *Vogtnema* with four lips; and *Hippopotamenema* has the oral opening directed ventrally and lacks lips. Thus, *Acanthoatractis* n. gen., n. sp. belongs to the first group of genera in which the oral aperture has sclerotized structures.

*Acanthoatractis* n. gen. has eight wrench-shaped sclerotized structures around the mouth. *Klossinemella* and *Orientatractis* differ from the new genus by the number and shape of sclerotized structures around the mouth. *Klossinemella* has an oral opening surrounded by two bilobed formations (dorsal and ventral) resembling lips, with eight Y-shaped sclerotized structures, two subdorsal pairs, two laterodorsal pairs, two subventral pairs, and two lateroventral pairs. Additionally, a single horn is present between each subventral pair, and every two pairs have two small sublateral processes resembling papillae, sclerotized and oriented posteriorly to the amphids. *Orientatractis* species have six lips; each submedian lip has a well-sclerotized, recurved, pointed, bicornate chitinous piece and a single median spine. Furthermore, all species of *Klossinemella* and *Orientatractis* have a gubernaculum (Moravec and Thatcher, 1997; Jesus et al., 2023), whereas *Acanthoatractis* n. gen. lacks a gubernaculum.

The new genus lacks papillae at the cephalic end, and the oral opening has two sclerotized pieces, each with a pair of median hooks (one dorsal and one ventral). *Crossocephalus*, described in equines and rhinoceros, can be easily distinguished from the new genus *Acanthoatractis* by its numerous papillae at the cephalic end. Moreover, *Crossocephalus* has three lips (one dorsal and two submedian), each lip with a pair of hooks, and the anterior end of the esophagus with three pairs of eversible pectinate blades. The excretory pore is post-bulbar, and the esophageal bulb has valves (Berenger, 1947; York and Southwell, 1920), while in *Acanthoatractis* n. gen. the excretory pore is pre-bulbar, and the esophageal bulb does not have valves.

In *Acanthoatractis* n. gen., lips are absent, displaying eight pairs of spines (two subdorsal pairs, two dorsolateral pairs, two subventral pairs, and two ventrolateral pairs), and spines are absent posterior to the amphid pores. In contrast, *Paraorientatractis* from freshwater turtles has an oral opening with four submedian lips, each lip bearing a pair of recurved spines fused at the base and a single median spine near the distal margin, along with a pair of smaller spines posterior to the amphids. Additionally, *Paraorientatractis* has cuticular projections along the dorsal surface of the body (Gibbons et al., 1997), while *Acanthoatractis* n. gen. has a smooth cuticle.

*Cobboldina* found in hippopotami (Mammalia: Hippopotamidae), has a mouth surrounded by a cuticular sheath with a triangular median extension in dorsal and ventral positions (Leiper, 1911; Mondal and Manna, 2012) and differs from the new genus by the absence of hooks or spines in the mouth.

Finally, the new taxon differs by having the cephalic end surrounded by 16 pairs of spines distributed in a circle in subdorsal, dorsolateral, subventral, and ventrolateral positions. *Grassenema*, described in hiracoids (Mammalia: Hyracoidea), has the cephalic end surrounded by 12 pairs of cuticular spines arranged in two circles: the anterior circle with four pairs in subdorsal and subventral positions, and the posterior circle with eight pairs in subdorsal, subventral, and sublateral positions. Additionally, *Grassenema* possesses a buccal capsule and pharynx (Saito et al., 2021), structures that are absent in *Acanthoatractis* n. gen.

Thus, based on these morphological characters, the nematodes described here represent the 27th genus among atractids and the seventh report of this family in the *P. unifilis* species.

## Ethics approval

We followed all applicable institutional, national, and international guidelines for animal care and use. The study is registered with the Animal Ethics Committee from the UFPA under code 8341260821. The present study was approved by Instituto Chico Mendes de Conservação da Biodiversidade (ICMBio), Brazil, and host specimens were collected under license number SISBIO: 53527-4.

## Funding

This study was supported by Coordination for the Improvement of High Higher Education Personnel (CAPES)/Postgraduate Program in the Biology of Infectious and Parasitic Agents (PPGBAIP)/UFPa, Amazon Foundation for Research and Studies Support (FAPESPA), Programa de Apoio a Nucleos Emergentes (FAPESPA/CNPq PRONEM 01/2021, process number 51/2021); Research productivity scholarship of CNPq to F.T.V. Melo (Process number 314116/2021-4). This study is part of the Ph.D. thesis of Ronald Ferreira de Jesus in PPGBAIP (Institute of Biological Sciences-UFPa).

## CRediT authorship contribution statement

**Ronald Ferreira Jesus:** Writing – review & editing, Writing – original draft, Software. **Bianca Nandyara:** Resources. **Jeannie Nascimento dos Santos:** Formal analysis. **Francisco Tiago de Vasconcelos Melo:** Writing – original draft, Supervision, Funding acquisition.

## Declaration of competing interest

The authors declare that they have no known competing financial interests or personal relationships that could have appeared to influence the work reported in this paper.
